# Feedback based on experience sampling data: Examples of current approaches and considerations for future research

**DOI:** 10.1016/j.heliyon.2023.e20084

**Published:** 2023-09-12

**Authors:** Sara Laureen Bartels, Catherine van Zelst, Bernardo Melo Moura, Naomi E.M. Daniëls, Claudia J.P. Simons, Machteld Marcelis, Fionneke M. Bos, Michelle N. Servaas

**Affiliations:** a Department of Psychiatry and Neuropsychology, School for Mental Health and Neuroscience, Maastricht University, Maastricht, the Netherlands; b Alzheimer Centre Limburg, School for Mental Health and Neuroscience, Maastricht University, Maastricht, the Netherlands; c Department of Psychosis Research and Innovation, Parnassia Psychiatric Institute, The Hague, the Netherlands; d GGzE Institute for Mental Health Care Eindhoven, Eindhoven, the Netherlands; e Institute of Pharmacology and Neurosciences, Faculty of Medicine, University of Lisbon, Lisbon, Portugal; f Universidade Católica Portuguesa, Faculdade de Medicina, Portugal; g Department of Family Medicine, Care and Public Health Research Institute, Maastricht University, Maastricht, the Netherlands; h Department of Psychiatry, Interdisciplinary Center for Psychopathology and Emotion Regulation, University of Groningen, University Medical Center Groningen, Groningen, the Netherlands; i Department of Psychiatry, Rob Giel Research Center, University of Groningen, University Medical Center Groningen, Groningen, the Netherlands

**Keywords:** Experience sampling method, Ecological momentary assessment, ESM-based feedback, Momentary data, Guidelines

## Abstract

Methodologies such as the Experience Sampling Method (ESM) or Ecological Momentary Assessment allow the gathering of fine-graded, dynamic, personal data within a patient's daily life. Currently, it is studied whether feedback based on experience sampling data (ESM-based feedback) can be used as a clinical tool to inform shared decision-making in clinical practice. Although the potential of feedback is recognized, little is known on how to generate, use, and implement it. This article (i) presents n = 15 ongoing ESM projects within the Belgian-Dutch network for ESM research wherein ESM-based feedback is provided to various patient populations, and (ii) summarizes qualitative data on experiences with ESM-based feedback of researchers (n = 8) with extensive expertise with ESM (average of 10 years) involved in these ongoing studies. The following aspects appear to be of relevance when providing ESM-based feedback: training for healthcare professionals and researchers, the use of online interfaces and graphical visualizations to present data, and interacting with patients in a face-to-face setting when discussing the contextual relevance and potential implications. Prospectively, research may build on these aspects and create coherent consensus-based guidelines for the use of ESM-based feedback.

## Highlights

• ESM-based feedback has a wide applicability in clinical research and clinical mental health.
• Guidelines for training to provide and interpret ESM-based feedback are needed and should be made publicly available.
• Personalization, understandability, and accessibility of ESM-based feedback seems key in effectively providing feedback.
• It is preferable to combine technology-based ESM with face-to-face feedback sessions to facilitate joint interpretation of the data.

## 1 Introduction

The use of experience sampling data in the realm of mental health and well-being is gathering momentum in research and clinical practice. Methodologies for self-monitoring, known as Ecological Momentary Assessment (EMA) [1] and the Experience Sampling Method (ESM) [2], enable the collection of momentary data that can provide information on contexts in which psychological symptoms occur, and factors promoting self-management and resilience [3]. Three of the main advantages of ESM are i) systematically informing patients and healthcare professionals (HCPs) on micro-level psychopathological processes of patients, ii) the possibility of collaboratively discussing this information and iii) making shared decisions based on this information about interventions [4].

A recent qualitative study on the use of ESM for psychiatric settings shows that HCPs and patients believe that ESM can support every phase of care, by enhancing a patient's self-awareness, insight, and self-management [5]. According to the social cognition theory, self-monitoring of own behaviors, its determinants, and effects influences and regulates an individual's thoughts, affect, and actions [6]. Hence, repeatedly completing ESM questionnaires in itself, and in combination with self-reflections or personalized feedback based on a patient's own experience sampling data, can increase self-awareness and improve coping [7]. This personalized feedback based on experience sampling data is here after referred to as ‘ESM-based feedback’.

Feedback based on experience sampling data may, for instance, be provided with a focus on positive affect (PA) [8]. According to the broaden-and-build-theory, a focus on PA can increase resilience and emotional well-being by broadening an individual's momentary thought–action repertoire and building personal resources [9]. Similarly, cognitive behavioral treatment approaches based on the psychological flexibility model rely on awareness as a core process for behavioral change [10], which may be facilitated through ESM-based feedback. Following, several studies have proven the effectiveness of ESM-based feedback in intervention studies to improve health care outcomes, such as depressive symptoms in participants (i.e., patients with depressive symptoms, caregivers of people with dementia) [11,12]. In these studies, personalized ESM-based feedback was discussed face-to-face to highlight daily situations in which participants engaged in activities that were related to greater levels of PA [11,12].

In clinical practice, ESM is described as a valuable tool for HCPs, and as empowering for patients [4,13]. For instance, people with early psychosis can benefit from ESM's nature of functional contextualism to provide and integrate Acceptance and Commitment Therapy in everyday life [14]. In psycho-oncological care, both cancer patients and HCPs experience self-monitoring and personalized feedback as helpful to gain insight into chronic cancer-related fatigue and to facilitate case conceptualization [15]. Moreover, patients with depression or bipolar disorder as well as their HCPs highlight that ESM with personalized feedback is insightful and contributes to self-awareness and self-management, even if behavioral changes or changes in symptomatology are not directly visible in clinical trials [[16], [17], [18]].

Despite its undeniable importance, the feedback process using ESM in mental health care is still understudied with limited research on this topic. It remains unclear how and which types of feedback are currently used. Moreover, it is an inherently complex matter that raises challenging methodological, practical, and ethical questions. Synthesizing characteristics of ongoing studies providing ESM-based feedback in mental health care research and clinical practice and qualitative information on initial experiences from ESM researchers is a first step towards urgently needed guidelines.

In this article, we (i) collect descriptive information on ongoing studies within the Belgian-Dutch network for ESM research providing ESM-based feedback to various patient populations aiming to provide a comprehensive overview of the contexts (e.g., population, outcomes, settings) and used feedback processes (e.g., frequency, length of session, content or focus of feedback, included parties, and training for feedback providers). Moreover, we (ii) qualitatively summarize experiences and insights (e.g., reasons for providing feedback, issues with or adjustments to the feedback process) across this network on providing ESM-based feedback with the aim to establish a first list of recommendations for ESM-based feedback in mental health care research and clinical practice.

## 2 Material and methods

### 2.1 Origin of the research idea

The research aims of this study originated from a ‘hackathon’ that took place during the ESM Expert Network Meeting in October 2019, Tilburg, the Netherlands, initiated by one of the authors (BM). The meeting was organized by the Belgian-Dutch Network for ESM research (https://esm-network.eu/), a collaboration between five universities in Belgium and the Netherlands, which have extensive experience with conducting ESM studies for more than twenty years. This network facilitates the increase of knowledge about ESM in mental health care research and promotes collaboration and innovation. During the ‘hackathon’, methodological, practical, and ethical aspects on providing ESM-based feedback in mental health care research and clinical practice were discussed. About twenty ESM researchers participated in the workshop (no fixed registration), and shared current practices and experiences including ‘what works best and what difficulties are faced when providing ESM-based feedback’. After this initial meeting, this discussion was followed up by the authors of this study.

### 2.2 Identification of ongoing studies

To fulfill the abovementioned research aims, an overview was created of ongoing studies providing ESM-based feedback within the Belgian-Dutch Network for ESM research (October 2019 n = 10 studies, updated in October 2022 with n = 5 additional studies). Studies were identified by contacting clinical coordinators and researchers from the Belgian-Dutch Network for ESM research via emailing lists and social media groups (i.e., network Facebook group), and asking whether the members of the network were performing a study fulfilling the following criteria: i) ongoing, ii) ESM-based feedback provided to participants/patients, iii) clinical research study.

### 2.3 Collection of descriptive information

For the first research aim (i.e., detailed overview of current ESM-based feedback studies), principal investigators (PIs) of the ongoing studies within the Belgian-Dutch Network for ESM research were asked to provide descriptive information about the study by completing two tables. The first table covered general study characteristics (i.e., study aim, design, population, setting, primary objective, and primary outcome measures). The second table covered details on the feedback process (e.g., digital interface, PDF, paper report etc.), ESM software/app used, face-to-face meeting (yes/no answer), length of feedback in minutes, frequency, timing, content, included parties, training, effectiveness (including both effectiveness of feedback alone or as part of a more elaborate intervention), and impact or burden of feedback. The tables were sent out as a Word document to the PIs of the identified ongoing studies (October 2019 and October 2022) via email.

### 2.4 Survey

To support the research aim (i.e., list of recommendations for ESM-based feedback based on ESM researchers' experiences and insights in mental health care research and clinical practice), a survey with ten questions was compiled to ask for qualitative information on the theoretical considerations for and practical experiences with providing ESM-based feedback. Open-ended questions included, for instance, ‘How or why did you choose the way you are providing feedback in your study?‘, and ‘Describe your positive/negative experience when giving feedback based on ESM data in your study’. Moreover, the respondents were asked for their current position (multiple-choice) and level of experience with ESM and clinical work (in years). The full survey can be found in Appendix 1.

Qualitative data helps to explore the context of and generate ideas for future studies [19]. The questions in this survey were based on discussions from the ‘hackathon’ and consensus meetings between the authors. The survey was sent out as a Word document to the PIs of the identified ongoing studies (October 2019), including two reminder emails. Survey respondents completed the survey digitally (i.e., in the Word document) and sent it back to SB or MS. Respondents are here referred to as ‘ESM experts’, namely researchers with theoretical and practical experience in planning and executing at least one ESM study wherein ESM-based feedback was provided in the context of mental health research or clinical practice.

### 2.5 Analysis of descriptive and survey data

Data related to the ongoing studies (i.e., first research aim) were analyzed descriptively, including sample sizes where suitable (i.e., n, range). Additional statistical analysis (e.g., means, SD) was not appropriate due to the small sample size or qualitative nature of responses.

Survey replies were merged into an Excel table. Qualitative data was then narratively summarized by two researchers (SLB, MNS), and findings were checked by a third researcher for completeness (BM). A narrative synthesis is a textual approach used to summarize and explain qualitative information according to themes [20,21]. In the present study, the narrative synthesis followed the survey's structure: ‘reasoning behind feedback approach’, ‘practical experience’, ‘issues’, ‘adjustments’, and ‘recommendations’. Based on this synthesis, a list of recommendations for ESM-based feedback processes in mental health care research and clinical practice was created. The list of recommendations was discussed with all authors until a consensus was reached.

### 2.6 Ethical statement

Study procedures were approved by the Medical Ethics Committee of the University Medical Center Groningen (METC UMCG; M22.306083). All participants signed an informed consent sheet.

## 3 Results

### 3.1 General characteristics of ongoing ESM projects

Descriptive information of n = 15 ongoing ESM studies was collected (Table 1). Studies differ in design (e.g., longitudinal observational study, randomized controlled trial, action research) and clinical settings (from primary to tertiary care). Target populations include, for instance, people with mental health issues (e.g., psychosis, bipolar disorder, anxiety) and neurological conditions (e.g., stroke, cognitive impairments) with a wide age range (e.g., adolescents, older adults >60 years). Studies focus on feasibility, usability, effectiveness, and implementation processes, applying both qualitative (i.e., interviews) and quantitative methods (i.e., questionnaires).

**Table 1:** General descriptive information on ongoing ESM-based feedback studies in the Belgian-Dutch Network for ESM research.

**#**	Study/project name	PI/project lead	Aim	Study design(s)	Study population	Setting	Primary objective	Primary outcome measure
**1**	CSP-DNA	B. Montagne	Evaluating feasibility and usability of using personalized ESM-based feedback on cognitive-behavioral patterns based to improve traditional joint crisis plans for individuals with borderline personality disorder	Pilot study	Borderline personality disorder	Psychiatric care	Assessing feasibility and usability of personalized ESM-based feedback	Qualitative interviews
**2**	DAILY	G. Kiekens	Developing individual risk monitoring models for non-suicidal self-injurious thoughts and behaviors and facilitate clinical management by providing insight in daily life functioning and relevant patterns of risk and behavior	Longitudinal observational study	HCPs and patients receiving mental health care	Secondary and tertiary care	Predicting individual risk	Self-injurious thoughts, urges and behaviors
**3**	ESM BD	M. Wichers	Investigating the utility of ESM and personalized feedback for the treatment of bipolar disorder	Qualitative study	Bipolar disorder	Tertiary care	Assessing patient and HCP perspectives on clinical utility of ESM	Qualitative interviews
**4**	ESM-MSD	M. Marcelis, R. Bakker	Investigating whether ESM, integrating psychopathological symptoms, cognitive and motor-sensory tasks, is a useful diagnostic and prognostic tool in the context of early psychosis	Longitudinal observational study	First episode psychosis	Secondary and tertiary care	Assessing ESM data utility as a diagnostic and prognostic tool	ESM measures of psychotic experiences, positive and negative affect, motor and cognitive function
**5**	ESM PSY	M. Wichers	Investigating the utility of ESM and personalized feedback for the diagnostics of first episode psychosis	Qualitative implementation study	First episode psychosis	Tertiary care	Assessing HCP perspectives on clinical utility of ESM as a diagnostic instrument	Qualitative interviews
**6**	iCASE	M. Zuidersma, R.C. Oude Voshaar	(1) Evaluating daily personal predictors of depressive symptoms and mood in older persons with depressive symptoms and cognitive impairments. (2) Investigating the presence, nature and direction of the daily temporal association between depressive symptoms, cognitive performance and sleep in older individuals. (3) Evaluating the feasibility, usability and clinical value of ESM combined with wearables in older persons with depressive symptoms and cognitive impairment	Observational single-subject study design	Persons≥60 years with depressive symptoms and cognitive impairments	Psychiatric care and neurology (memory clinics)	Identifying daily triggers of depressive symptoms	Depressive symptoms
**7**	IMPACT	I. Myin-Germeys	Investigating (1) implementation feasibility of ACT-DL in Flemish routine mental healthcare, (2) the clinical feasibility and usability of ACT-DL, (3) its preliminary effectiveness, and (4) its generalizability across multiple settings	Pragmatic implementation pilot	Population with mental health problems	Secondary and tertiary care	Assessing feasibility and usability of ACT-DL	Adapted version of the MAUQ (includes usability and acceptability of the intervention) – therapist and client version Qualitative interviews
**8**	IMPROVE	I. Myin-Germeys	Evaluating the feasibility of implementing ESM in the clinic	Pilot study	HCPs and patients receiving mental health care	Tertiary care	Assessing feasibility of implementing ESM in the clinic	Adapted version of the MAUQ (including usability) Qualitative interviews
**9**	LIME	P. Delespaul, A. Beurskens, M. van Bokhoven	(1) Investigating how ESM can be embedded as an mHealth tool to support functional analyses in referred patients with anxiety or sleeping problems in family medicine. (2) Investigating experiences from patients and their mental HCPs concerning the use of ESM	Action research	Patients with anxiety or sleeping problems Psychological assistants to the general practitioner	Primary care (family medicine)	Assessing patient and mental HCP perspectives on the use of ESM	Qualitative interviews
**10**	Moe-*i*-teloos	C. van Heugten	Evaluating the (cost-)effectiveness of a 6-week ESM and feedback intervention for fatigue after brain injury	Non-randomized controlled trial	Stroke or traumatic brain injury	Primary (occupational therapy practice), secondary (hospital treatment), and tertiary care (rehabilitation center)	Decreasing fatigue	Fatigue Severity Scale
**11**	N1AP	C. Simons, M. Marcelis	(1) Evaluating the consequences of dose reduction of antipsychotic medication by detecting meaningful within-subject changes in daily life mental states that occur during and after dose reduction. (2) Determining the clinical effects of dose reduction of antipsychotic medication under longitudinal ESM self-monitoring by meta-analyzing 30 N = 1 trials to investigate aggregated-level trends in the effects of dose reduction	Multiple (n = 30) N = 1 trials	Psychotic disorder	Secondary and tertiary care	Dose-optimization	ESM measures of psychotic experiences, subjective wellbeing, social functioning, cognition, sleep, dopamine super-sensitivity, negative symptoms
**12**	PETRA	H. Riese, M. Wichers	Implementing ESM in psychiatric care by developing an intuitive web-based interface that helps to design a personalized ESM diary and automatically provides dynamical visual feedback	Implementation study	Transdiagnostic	Secondary and tertiary care	Developing a clinical personalized ESM tool	Design thinking, qualitative interviews, evaluation questionnaires
**13**	SELFIE	U. Reininghaus	Investigating the efficacy of a novel, accessible, transdiagnostic ecological momentary intervention (EMI) for improving self-esteem (‘SELFIE’) in youth aged 12–26 with prior exposure to childhood adversity	RCT	Youth (12–26 y.o.) with prior exposure to childhood adversity	Secondary care and general population	Improving self-esteem	Rosenberg Self-Esteem Scale
**14**	Therap-i	H. Riese	Investigating the efficacy of personalized ESM and ESM-based feedback (Therap-i module) integrated in outpatient psychological treatment for depression (treatment as usual)	RCT	Major depressive disorder	Tertiary care	Decreasing depressive symptoms	Inventory of depressive symptomatology (IDS-SR)
**15**	ZELF-i	J.A. Bastiaansen	Investigating the efficacy of self-monitoring and personalized feedback as an add-on tool in the treatment of depressive complaints in a natural setting	RCT	Major depressive disorder	Secondary care	Decreasing depressive symptoms	Inventory of depressive symptomatology (IDS-SR)

ACT-DL: Acceptance and Commitment Therapy in Daily Life. ESM: experience sampling method. NA: not applicable. PI: principal investigator. RCT: Randomized controlled trial. MAUQ: mHealth App Usability Questionnaire. HCP: healthcare professional. Note: This overview was last updated in October 2022.

### 3.2 Feedback procedures in ongoing ESM projects

For details of the feedback procedures, see Table 2. Feedback is usually provided using a digital interface (n = 14), while n = 1 project uses a paper-format. RoQua (www.roqua.nl) (n = 7), the PsyMate™ (www.psymate.eu) (n = 5), and m-path (www.m-path.io) (n = 3) are the used platforms. Frequency and timing of feedback sessions vary, from one session at the end of the study period to an unspecified amount (as determined by patients and HCPs). Next to descriptive graphs or diagrams (i.e., pie charts, bars, line graphs) used in all studies, one project additionally includes dynamic time-series models via ESMvis, a framework for providing descriptive feedback, focusing on direct visualization of the dynamic nature of raw data, and one study applies person-specific network models. In all projects, feedback is provided face-to-face. Face-to-face sessions ranged in length from 15 min to 2 h. Most projects (n = 12) include a workshop/training to prepare HCPs or researchers to provide feedback, however, in some cases this training is optional (n = 3). In the paper-based feedback study, no training is included. Impact or burden of the feedback on patients is evaluated using qualitative interviews or evaluation questionnaires.

**Table 2:** Descriptive information on ESM-based feedback of ongoing ESM-based feedback studies in the Belgian-Dutch Network for ESM research.

**#**	Study/project name	Form	Feedback/ESM Software	Face-to-face meeting	Length of feedback	Frequency	Timing	Content	Included parties	Training	Effectiveness^1^	Impact/Burden
**1**	CSP-DNA	Digital movie and poster	RoQua/ESMvis visual feedback using R	Yes	Circa 60 min.	1	After 3 or 4 weeks of ESM monitoring	Text and descriptive graphs + ESMvis poster + ESMvis descriptive movie	Patient, HCP and researcher	Pilot, training will be developed	Qualitative interviews with patients	Qualitative interviews with patients
**2**	DAILY	Digital report via e-mail	m-Path/m-path	Yes	50 min.	1	After 4 weeks of ESM monitoring	Descriptive graphs	Patient and HCP	For HCPs, an online training (optional)	Self-injurious thoughts, urges, and behaviors	Qualitative interviews with patients and HCPs and evaluation questionnaires filled in by patients and HCPs
**3**	ESM BD	Digital report via e-mail	RoQua/RoQua	Yes	40–60 min.	1	After 4 months of ESM monitoring	Descriptive graphs	Patient, HCP and researcher	For HCPs, a training on how to interpret the graphs and how to structure the discussion with the patient	Qualitative interviews with patients and HCPs	Qualitative interviews with patients and HCPs
**4**	ESM-MSD	Digital feedback interface	PsyMate™/PsyMate™	Yes	15 min (and open)	3	At the end of 3 ESM waves of the study	Descriptive graphs	Patient and researcher	NA	NA	NA
**5**	ESM PSY	Digital report via e-mail	RoQua/RoQua	Yes	Flexible (integrated in diagnostic advice meeting)	1	At the end of the diagnostic phase	Descriptive graphs, dependent on relevant clinical questions	Patient and HCP (psychiatrist and psychiatrist in training, psychiatric nurse)	For HCPs, a training on how to interpret graphs as diagnostical instruments	Qualitative interviews with HCPs	Qualitative interviews with HCPs
**6**	iCASE	Paper report	In-house SPSS and R script/RoQua	Yes	1–2 h	1	After the study ends	Descriptive(s) graphs and temporal associations with VAR analysis	Patient, HCP, researcher	NA	Depressive symptoms	Evaluation questionnaires filled by patients
**7**	IMPACT	Online dashboard	m-Path/m-Path	Yes	Flexible (determined by the HCP, 8 ACT therapy session)	Circa 8 times (depends on total number of ACT therapy sessions provided)	Depends on time between therapy sessions; every 1–2 weeks (after each ACT session in combination with ESM monitoring)	Descriptive graphs, word cloud (topic of value description within ACT therapy)	Patient and ACT-DL HCP	For HCP, an online training on how to work with the dashboard and a manual wherein the therapy ACT-DL protocol is described	Insight and self-reflection; ACT coping skills; client empowerment and disease management; symptoms, distress, wellbeing and quality of life Qualitative interviews with patients and HCP	Adapted version of the MAUQ (including usability and acceptability of the intervention) – therapist and client version Qualitative interviews with patients and HCP
**8**	IMPROVE	Online dashboard	m-Path/m-Path	Yes	50 min.	1	After 1 week of ESM monitoring	Descriptive graphs	Patient and HCP	For HCP, an online training and manual (optional)	Compliance, Self-reflection and insight, self-management, self-efficacy, therapeutic self-care, patient activation, service engagement, work alliance, shared-decision making, depression, anxiety and stress Qualitative interviews with patients and HCP	Adapted version of the MAUQ (including usability) Qualitative interviews with patients and HCP
**9**	LIME	Digital feedback interface	PsyMate™/PsyMate™	Yes	Circa 35 min.	Flexible (determined by the psychological assistant to the GP, in dialogue with the patient)	Flexible (determined by the psychological assistant to the GP, in dialogue with the patient)	Descriptive graphs and diagrams	Patient, psychological assistant to the GP, and sometimes researcher	For assistants to the GP, a training and work instruction about the use of ESM technology to support detailed functional analyses within their work processes. For patients, an instruction about the download, login procedure, and use of the tool	NA	Qualitative interviews with patients and the psychological assistants to the GP
**10**	Moe-*i*-teloos	Digital feedback-interface	PsyMate™/PsyMate™	Yes	45–60 min.	6	After each week of ESM monitoring	Descriptive graphs	Patient and HCP	For HCP, a workshop on how to use the graphical PsyMate interface to provide feedback on ESM data	Fatigue, cognitive and emotional symptoms, personal goals, societal participation, cost-effectiveness	Evaluation questionnaire filled in by patients
**11**	N1AP	Digital report including most relevant graphs and written evaluations of the feedback session	PsyMate™/PsyMate™	Yes	45 min	After each ESM week or twice weekly, ESM monitoring takes place circa 16 weeks plus 6 monthly FUs plus 3 yearly FUs	After each week of ESM monitoring or twice weekly	Descriptive graphs	Patient and HCP/researcher	For HCPs or researchers on how to use the feedback module and on how to provide feedback	Momentary psychotic experiences, subjective well-being, social functioning, cognition, sleep, dopamine super-sensitivity, negative symptoms	Evaluation questionnaire filled in by patients
**12**	PETRA	Digital feedback interface and digital report	RoQua/RoQua	Yes	45–60 min	Flexible (determined by the HCP and patient)	Flexible (determined by the HCP and patient)	Descriptive and interactive graphs	Patient and HCP	For HCPs, a workshop on how to use the PETRA interface	Qualitative interviews and usability sessions with patients, and evaluation questionnaires filled in by patients	Qualitative interviews and usability sessions with patients, and evaluation questionnaires filled in by patients
**13**	SELFIE	Digital report	NA/PsyMate™	Yes	45–60 min	3 face-to-face 3 emails	Face-to-face: week 1, 3, 5 Email: week 2, 4, 6	Feedback on their use of the exercises in the app	Participant and HCP	For HCPs, a training and guideline with detailed instructions on how to provide the intervention	NA	Evaluation questionnaires filled in by participants
**14**	Therap-i	Digital feedback interface and digital report, including discussed graphs and notes, sent via email	In-house R script/RoQua	Yes	45–60 min	3	Week 2, 4 and 8	Descriptive and network graphs on cognitions, emotions, behavior, body and context	Patient, HCP and researcher	For HCPs, a workshop is offered on how to use the Therap-i module in their treatment. Participation is not necessary to undergo the ESM module.	Depressive symptoms, psychological functioning, illness perception, therapeutic alliance, self-management	Evaluation questionnaires filled in by patients and HCPs, and a qualitative interview with a subset of patients
**15**	ZELF-i	Digital report via e-mail	In-house R script/RoQua	Yes	45–60 min	4 reports, 1 meeting	Week 1, 2, 3, and 4	Text and descriptive graphs and (after week 4) temporal associations with VAR analysis between sets of variables (i.e., affect and activities or thinking patterns)	Patient and researcher	For research assistants a training on how to support patients in interpreting the graphs	Depressive symptoms, psychosocial functioning and feelings of empowerment	Evaluation questionnaires filled in by patients and a qualitative interview with a subset of patients

ACT: Acceptance and Commitment Therapy. ACT-DL: Acceptance and Commitment Therapy in Daily Life. ESM: Experience sampling method. FU: follow-up. GP: general practitioner. HCP: healthcare professional. MAUQ: mHealth App Usability Questionnaire. NA: not applicable. PETRA: PErsonalized Treatment by Real-time Assessment; VAR: vector autoregression. ^1^Note: Effectiveness may refer to the effects of the feedback alone or as part of a more elaborate intervention (e.g., Therap-i module includes resilience interview, ESM monitoring, and ESM-based feedback). This overview was last updated October 2022.

### 3.3 Characteristics of ESM experts

Six questionnaires were filled in by n = 8 researchers (survey could be completed in collaboration). Survey respondents were PhD students (n = 3), postdoctoral researchers (n = 2), or senior researchers (n = 3) with an average of 10.56 years of experience with ESM (range 3.5–35 years).

### 3.4 Reasoning behind feedback approaches

Researchers based their decision for the feedback approach on several arguments. First, previous research designs of own or other ESM studies were followed. Second, the feedback approach was guided by own expertise and knowledge build over the years related to ESM in general and its use in care processes, specific diseases and conditions, as well as knowledge related to cognitive behavioral treatment mechanisms. Third, practical arguments were mentioned as certain digital platforms or feedback forms (e.g., digital) were available and appeared suitable. Fourth, the way feedback was provided was informed in a co-creation process with HCPs. Finally, approaches also appeared to evolve as novel insights were gained during the study and thus, novel styles of providing ESM-based feedback were applied (e.g., a design with the main goal to collaboratively explore the results).

### 3.5 Practical experience with providing ESM-based feedback

Researchers highlighted the following benefits for patients. First, ESM-based feedback can help to gain (new) insights into own experiences or validate known patterns. It appeared helpful to patients to see lived experiences reflected in the ESM data. Sometimes ESM-based feedback was seen as “evidence” by patients confirming their experiences during daily life and treatment. Even if anticipated treatment outcomes might not be achieved, patients may feel that they gained a better control over their life through the ESM-based feedback sessions. Second, ESM-based feedback can lead to feelings of empowerment in patients as they are actively involved in the treatment process. Third, the ESM data and feedback sessions can have a positive influence on the interaction between the patient and HCPs and thus, sometimes improved therapeutic alliance. Specifically, survey responders indicated that ESM-based feedback may provide relevant input for shared interpretation and care decisions. Benefits for HCPs were that ESM-based feedback can help set the agenda for the session and guide treatment directions.

Researchers also mentioned drawbacks of ESM and ESM-based feedback. First, the ESM data collection process may not be suitable for every patient. A lack of motivation or difficulties managing technology can result in dropouts or too little data. Particularly when symptoms are severe, ESM may be experienced as too burdensome. Second, data sets might not show fluctuations because symptoms are at a consistent level, or patients hesitate to report fluctuations, meaning that no patterns can be discussed, and ESM-based feedback may not be helpful. Third, patients can misunderstand the feedback information, especially network graphs. It is therefore important to carefully explain the meaning and limitations of (visual outputs of) feedback information. Fourth, patients can be overstimulated during feedback sessions when data sets are extremely rich and clear take-home messages are lacking. The latter might be particularly difficult for HCPs who are less experienced with ESM or related statistics. Tying data to clinical conclusions can be challenging. Some HCPs might, for instance, hope that data results in clear-cut answers to patient's problems, which can result in disappointment, as researchers indicated.

### 3.6 Issues with patients’ understanding, engagement with, and action on feedback

While most patients face no problems in relation to ESM-based feedback, potential issues can appear, according to researchers. A first issue might lie in *understanding feedback*; while graphs and figures can be helpful, they can still be (too) complex. A certain degree of digital skills appears necessary to interact with the feedback page and sensory handicaps (e.g., visual impairments) require novel forms of data representation. Verbal explanations can be useful to clarify graphs and figures. Specifically, detailed elaborations on graphs and statistics (e.g., What is a “mean”?), also adjusted to the patient's level of education, may be necessary. Furthermore, causal interpretations, which patients might wish for, cannot be made as correlations do not equal causality. Especially complex associations or networks containing multiple associations between variables may not be understandable for some patients.

The second issue can lie in *engaging with feedback*; reflective questions (e.g., What does this information mean to you? How do you view this pattern?) were mentioned to facilitate the joint interpretation of feedback information. However, in certain populations, such as individuals who experience higher levels of negative (psychotic) symptoms, it was seen as complicated for HCPs to know how or if patients are engaged in sessions. Patients’ symptoms might influence attention and information processing, making it difficult to take in feedback.

Finally, *acting on feedback* was mentioned by researchers as the most challenging, and potentially least successful part of the process. When clear patterns emerge, patients may be advised to engage in certain activities more often. However, within research studies, evaluation of behavioral changes was often not part of the evaluation. If a patient received care, it was also recommended that insights are shared by the researcher with treating HCPs to support care processes.

### 3.7 Adjustments of providing ESM-based feedback during the study

In some cases, researchers indicated that feedback was always provided in the same manner. If adjustments were made, researchers learned by experience to include patients more in the conversation to make data interpretation less abstract, and HCPs learned over time how to discuss feedback by experiences with patients and coaching-on-the-job. Specifically, they learned to focus on a few main points while providing feedback, and to keep feedback as simple and accessible as possible, instead of going over all points that seem interesting.

### 3.8 Preliminary recommendations from ESM researchers

Several recommendations were provided by the ESM researchers based on theoretical considerations and practical experiences. These preliminary recommendations are presented below (Table 3).

**Table 3:** Preliminary recommendations for healthcare professionals (HCPs) and researchers when providing ESM-based feedback based on experts’ input.

• Personalize the feedback as much as possible, as not every person will find the same information interesting or relevant.
• If possible, prioritize topics of relevance to the patient/HCPs.
• Generate automated scripts to reduce burden for researchers/HCPs when processing information for feedback.
• Discuss feedback face-to-face and take adequate time to interpret the data together.
• Explore beforehand what HCPs and patients expect from the feedback to prevent misunderstandings or disappointment.
• Focus the feedback on a few main areas of interest.
• Engage patients in the feedback sessions by, for instance, asking questions and encouraging them to share their thoughts to create a collaborative care process.
• Ideally, feedback sessions take place during or directly after the self-monitoring (ESM data collection) period and are frequent (i.e., every or every other week); this might also enhance motivation for ongoing self-monitoring.
• Avoid causal interpretations of the data or viewing information without context.
• Explain graphs and data (e.g., What is a Mean?), keep feedback as simple as possible to improve understandability for patients, and ensure patients grasp the core message.
• Provide a feedback report including a summary and graphs; potentially share the report with treating HCPs.
• Keep in mind: ESM is just a tool and might not be able to replace face-to-face contact and counselling, particularly for patients who are struggling with self-management.

Note: These recommendations are based on a reflections from researchers with extensive ESM (n = 8, average: 10 years of working with ESM), but do not represent a complete consensus list.

## 4 Discussion

There is a need to improve our understanding of how ESM-based feedback can be optimally provided in both mental health research and clinical practice. The ongoing studies presented in this paper are examples of heterogeneous research trials in the Belgian-Dutch Network for ESM research covering a broad range of designs, target populations, settings, and aims, which speaks for a wide applicability of ESM-based feedback. Generally, ESM researchers performing these studies experienced feedback processes as feasible and beneficial. However, the evidence for assumptions underlying ESM-based feedback, specifically the increase in insight, self-awareness, and self-management are still limited, and require more research [22]. To this end, we encourage the global scientific community to critically review and build on the preliminary recommendations provided here to establish coherent guidelines prospectively. Additionally, several aspects stood out and are discussed: (i) training for providing and interpreting ESM-based feedback; (ii) technologies and forms of presenting ESM data; and (iii) technology-based data collection in combination with face-to-face feedback sessions: the blended care approach.

### 4.1 Training is key: but how?

Providing and supporting the interpretation of ESM-based feedback can be challenging, as the data is rich, and a methodological novice might feel overwhelmed or insecure about the process. Therefore, training seems essential to prevent inaccuracies, to enhance feelings of security and self-efficacy, and to utilize feedback to its full potential. Both ESM researchers and healthcare providers seem aware of this necessity [23]. The present study did not gather detailed information on how training was provided or what training comprised of, thus, is unable to provide a comprehensive answer. Moreover, method sections in scientific publications leave the question widely unanswered, only describing who is providing the feedback [11], or that ‘training with clear instruction on how to provide feedback’ was received (p.4) [24]. Similarly, while general guideline for briefing participants in ESM studies exist [25,26], how junior ESM researchers are trained to brief participants when installing the smartphone app and completing momentary assessments is not transparently described in studies.

In the eHealth literature, training has been highlighted as essential to interact with and benefit from technologies in healthcare [27,28]. To enhance the sustainable implementation of ESM-based feedback into healthcare, the set-up and provision of the training is ideally integrated into the procedures of clinics. Prospectively, the global ESM community including the Belgian-Dutch Network for ESM research and HCPs in the field need to provide detailed descriptions in publications, add manuals and instructions in appendices, contribute to the development and dissemination of guidelines [29], and evaluate the impact of training on ESM-based feedback.

### 4.2 Technologies to create personalized graphs, texts, and movies

The complexity of momentary data requires the exploration of appropriate and understandable means to present information to patients. Several tools are available in Belgium and the Netherlands to facilitate data collection, interpretation, and feedback processes. For example, at University Medical Center Groningen, The Netherlands, PETRA was developed in collaboration with patients and HCPs [30]. PETRA is a web-application that enables ESM monitoring and feedback in psychiatric care. It includes a decision aid to support patients and HCPs with constructing personalized ESM diaries, a diary item repository, and an interactive feedback module for visualizing the data. Feedback modules focus on themes identified by patients and HCPs as relevant to psychiatric treatment (e.g., fluctuations and changes in symptoms over time, associations between (social) contexts and symptoms). PETRA is integrated in the electronic health record system to ensure ease-of-use and sustainability. The effectiveness of adding personalized ESM-based feedback to depression treatment is currently evaluated in the Therap-i randomized controlled trial [31].

Another ESM technology that combines data collection and visualization is m-Path, a user-friendly online platform developed by KU Leuven, Belgium [32]. M-Path provides features for ESM monitoring and interventions, in which treatment content is accessible within the smartphone app. Results can be analyzed and visualized in timelines or weekly/daily patterns. The basic version of m-Path is freely available and premium features are optional [32]. Next to previous and ongoing studies in Belgium and the Netherlands, m-path is also used in studies abroad [33].

Finally, the PsyMate™ is an ESM platform developed by Maastricht University, the Netherlands. The clinical use of paper-pencil-based ESM in Maastricht started in the mid 80s. In the early 90s, and the first ESM feedback procedures were explored. Paper-pencil was then replaced by a palmtop computer, and later by a smartphone app offering momentary data collection and interpretation, facilitating the visualization of feedback via a web-based feature. This feature cannot be accessed via the app but requires a separate web-site login. Basic use of the PsyMate™ is free, and advanced packages are also available. The PsyMate™ has been used in many studies [11,12,[34], [35], [36]], also including the development and testing of cognitive momentary tasks [37,38].

In addition to all-in-one platforms, a specific feedback tool was developed that is not part of a data collection platform. ESMvis, developed at the University of Groningen, is a freely available framework turning raw data into dynamic personalized feedback (e.g., movies), including overall trajectories and specific time points [39]. Tested over 52 weeks in a patient with obsessive-compulsive disorder and their HCP, ESMvis was able to visually determine two relapses, making it an insightful add-on tool for clinical practice [39].

Finally, in addition to the platforms used in ESM studies in the Belgian-Dutch context, other instruments such as Moodbuster, Movisens, ilumivu, or Tempest are used [40,41]. Scientists and HCPs are encouraged to evaluate which ESM tool fits their specific purpose best, also taking patients’ abilities and needs into account. Practical reasons, such as costs and familiarity, will influence the choice, but ultimately, the patient should benefit most from the momentary data collection and feedback processes. The authors emphasize that there may not be a “best option” when deciding on a technology for ESM-based feedback, but the specific context determines optimal suitability. Nevertheless, transparency on why a certain technology was chosen and if the technology performed as expected should be enhanced.

### 4.3 Not losing the human touch: face-to-face feedback sessions

It may appear logical that digital data collection (i.e., completing questions in a smartphone app) would also result in digital feedback. When examining eHealth technologies, such as wearables monitoring step count or heart rate, a reporting page is usually part of the features, and the abovementioned ESM technologies also include reporting pages. Viewing own data in combination with automatic motivational messages are known as ‘just-in-time’ micro-interventions and can be helpful for enhanced self-management [42].

However, all included projects include face-to-face feedback sessions. This approach is different from self-management micro-interventions, and appears necessary for ESM data, as more complex health issues also require more guidance. Blended feedback and care – the combination of eHealth with face-to-face interactions – has been recommended, for instance for patients with a chronic illness [43]. Wentzel et al. (2016) further report that relevant requirements for blended care in mental health are that both modalities complement each other, and that the set-up of blended treatment is based on shared decision-making [44], which is also the common case in ESM-based feedback.

In the context of digital ESM interventions for middle-aged and older adults, it is suggested to combine face-to-face feedback sessions with automated feedback, to let individuals benefit from personal contact while also supporting them throughout daily life [45]. While this approach was not directly mentioned in ongoing studies, certain ESM technologies allow patients to view their own data, before having in-depth conversations with their HCPS face-to-face. Both the patient and HCP bring relevant expertise to the table, that is, the patient has knowledge about their inner world and background, whereas the HCP has knowledge about theories, research, and practice [46]. By collaboratively discussing ESM-based feedback, patient and HCP may gain more information to describe, explain, and better understand complaints and decide together which intervention to prioritize. In the end, technology cannot replace HCP-patient relationships, particularly in individuals who struggle with self-management, and therefore, a carefully designed blended care ESM-based feedback approach appears most beneficial.

### 4.4 ESM-based feedback as a reward for participation in research

Longitudinal ESM sampling can be used for research purposes only, without the aim to intervene on symptoms or behaviors. A person's motivation for repeatedly filling in daily assessments is then less intrinsic, as direct health benefits cannot be anticipated. ESM-based feedback can function as a reward to make participation in research more attractive and to enhance completion rates [47]. For instance, in an ongoing study, unstructured feedback based on participants' interests is provided at the end of the ESM period as a reward for study participation (Analysis in progress, Study protocol [48]). Another example of such feedback is the national study ‘How nuts are the Dutch’, in which participants (n = 12,503) received instant and automated feedback on their own data in comparison to the Dutch sample [49]. Next to these projects, more ESM studies might include feedback in such a way. If the study aim is clearly communicated and no ethical issues are present (e.g., participants expect health benefits), ESM-based feedback in itself can be a rewarding outcome for participants.

### 4.5 Limitations

This study does not aim to be exhaustive or a full consensus among the global ESM research community. Instead, we provide first recommendations for ESM-based feedback processes in mental health research and clinical practice based on expertise and insights within the Belgian-Dutch Network for ESM research. Thus, we would like to stress that the generalizability of the findings is limited and mainly reflects approaches with a certain geographical homogeneity, despite the reported heterogeneity of 15 included ESM studies. The sample of ESM experts that provided qualitative data is small (n = 8) and the qualitative information does not cover all trials presented in the tables, as some approached researchers did not complete the survey (reasons not provided for confidentiality purposes). Moreover, during the update (October 2022), PIs were only asked to provide descriptive information for the tables. Additionally, we did not collect information on how long it took participants to complete the survey as surveys were sent via email. Furthermore, the experiences and recommendations were assessed via a self-report questionnaire. Most probably, a semi-structured interview with the ESM researchers would have led to more detailed information. Finally, the quality of the presented studies was not determined and cannot be assessed as projects are still ongoing.

### 4.6 Future directions

Although ESM research has a long tradition in the Belgian-Dutch context [50] and survey respondents had extensive experience with performing ESM(-based feedback) studies, larger international studies, using methods to establish a full consensus agreement (e.g., Delphi technique) [51], will be useful to develop comprehensive guidelines. Such a trial and consensus should also consider the views and experiences of patients and other healthcare professionals. Future studies building on the present findings (specifically the preliminary recommendations) should further investigate the topic of providing ESM-based feedback and especially include scientific groups outside the Belgian-Dutch Network for ESM research.

Additionally, the focus of feedback using momentary data could be broadened to cover not only active ESM self-monitoring from digital diaries, but also passive ambulatory assessments/EMA (e.g., wearables, pedometers) with a focus on, for instance, physical outcomes [52], or a combination of both. To a certain degree, the mechanisms of both feedback processes as described in the social cognition theory (i.e., increased self-awareness and promotion of behavioral change) might overlap as it is not specified if self-monitoring must be active or passive [6]. Combining active and passive ESM data comes with specific challenges. In this regard, we would like to refer the reader to these references [53,54] to explore the topic further, of which one [55] is also an outcome of a hackathon of the Belgium-Dutch Network for ESM research. Prospectively, a growth of knowledge on link between active and passive monitoring is expected as some ongoing studies are collecting passive data (e.g., actigraphy data in the Therapi-study). Feedback on the combination of both remains in its infancy but holds promise for healthcare, especially in the context of just-in-time adaptive interventions [56,57].

## 5 Conclusions

This paper (i) presents an overview of ongoing studies in the Belgian-Dutch Network for ESM research providing feedback on experience sampling data in clinical populations and (ii) summarizes experiences and preliminary recommendations from ESM researchers involved in these studies. The ongoing ESM-feedback studies presented in this paper include examples of trials with a broad range of research designs, target populations, work settings, and objectives, which speaks for a wide applicability of ESM-based feedback. Experiences of researchers from these studies have indicated that training to provide ESM-based feedback is important as well as the establishment of guidelines and making them publicly available. Furthermore, as ESM data is rich, one needs to carefully consider how to present feedback visually and verbally, particularly for patients with complex mental health needs. We look forward to the outcomes of the ongoing ESM-based feedback studies and the specific role ESM-based feedback will play in the future of clinical mental health.

## Funding

No specific funding was received for this article.

## Author contribution statement

Sara Laureen Bartels, PhD; Michelle Servaas, PhD: Conceived and designed the experiments; Performed the experiments; Analyzed and interpreted the data; Contributed reagents, materials, analysis tools or data; Wrote the paper.

Catherine van Zelst, PhD: Conceived and designed the experiments; Performed the experiments; Wrote the paper.

Bernardo Moura: Conceived and designed the experiments; Analyzed and interpreted the data; Wrote the paper.

Naomi E.M. Daniëls, PhD; Claudia J.P. Simons, PhDORCID; Machteld Marcelis, PhD; Fionneke M. Bos, PhD: Conceived and designed the experiments; Wrote the paper.

## Data availability statement

Raw qualitative data cannot be shared due to confidentiality. All other data is transparently reported in Table 1, Table 2

## Declaration of competing interest

The authors declare that they have no known competing financial interests or personal relationships that could have appeared to influence the work reported in this paper.
