# Supplementary material for: Feedback based on experience sampling data: Examples of current approaches and considerations for future research
Source: Heliyon. 2023 Sep 12;9(9):e20084. doi: 10.1016/j.heliyon.2023.e20084 (PMC10559801; doi:10.1016/j.heliyon.2023.e20084)
Supplement: Multimedia component 1 [file mmc1.docx]

**APPENDIX.**

Appendix 1. Survey questions given to the researchers involved in the ongoing ESM studies providing feedback.

| 1 | Current position of person(s) filling in the survey (multiple-choice: PI, Postdoc, PhD student, Bachelor’s/ Master’s student, Other: please indicate). |
| --- | --- |
| 2 | What is your level of experience (i.e., yrs. of working as a clinician, yrs. of working with ESM)?^1^ |
| 3 | Please provide a short summary of any kind of script/ guideline/ instruction you may use when giving ESM feedback in your study.^1^ |
| 4 | How and why did you choose the way you are providing feedback in your study?^1^ |
| 5 | Please describe your positive experience when giving feedback on ESM data in your study.^1^ |
| 6 | Please describe your negative experience when giving feedback on ESM data in your study.^1^ |
| 7 | Please reflect on if and how the patients you are working with are able to understand, engage, and act on the feedback you provide.^1^ |
| 8 | Did your experience during your study change the way you provide feedback? If so, how?^1^ |
| 9 | We understand that your study is still ongoing. Nevertheless, we are interested in any recommendations you may have based on this study (and your previous experiences with ESM) when giving feedback in clinical mental health care/ research.^1^ |
| 10 | Would you like to add anything else about this topic? Did you miss anything in this questionnaire?^1^ |

*Note: ^1^These questions were open.*
